# A systematic review of population health interventions and Scheduled Tribes in India

**DOI:** 10.1186/1471-2458-10-438

**Published:** 2010-07-26

**Authors:** KS Mohindra, Ronald Labonté

**Affiliations:** 1 Institute of Population Health, University of Ottawa, Ottawa, Canada

## Abstract

**Background:**

Despite India's recent economic growth, health and human development indicators of Scheduled Tribes (ST) or *Adivasi *(India's indigenous populations) lag behind national averages. The aim of this review was to identify the public health interventions or components of these interventions that are effective in reducing morbidity or mortality rates and reducing risks of ill health among ST populations in India, in order to inform policy and to identify important research gaps.

**Methods:**

We systematically searched and assessed peer-reviewed literature on evaluations or intervention studies of a population health intervention undertaken with an ST population or in a tribal area, with a population health outcome(s), and involving primary data collection.

**Results:**

The evidence compiled in this review revealed three issues that promote effective public health interventions with STs: (1) to develop and implement interventions that are low-cost, give rapid results and can be easily administered, (2): a multi-pronged approach, and (3): involve ST populations in the intervention.

**Conclusion:**

While there is a growing body of knowledge on the health needs of STs, there is a paucity of data on how we can address these needs. We provide suggestions on how to undertake future population health intervention research with ST populations and offer priority research avenues that will help to address our knowledge gap in this area.

## Background

Despite India's recent economic growth, health and human development indicators of Scheduled Tribes (ST) or *Adivasi *(India's indigenous populations) lag behind national averages [1,2].(See Table 1) While there has been some improvement for STs, the gaps remain wide and for some indicators have even been increasing (Table 1). These disparities persist despite the Indian government's pursuit of affirmative action since independence and their investments in Tribal development programs [3]. There has been considerable debate on reservations of government seats, public jobs, and education institutions,[4] but the role of population health interventions in improving the situation for STs has received relatively little attention.

**Table 1:** Selected health indicators in India for total population and STs, 1992-2006

	**Total population**			**ST**		
		
**Indicator**	**1992-93**	**1998-99**	**2005-06**	**1992-93**	**1998-99**	**2005-06**
	
Infant mortality rate (per 1,000)	86.3	73.0	57.0	90.5	84.2	62.1
Under 5 mortality rate (per 1,000)	118.8	101.4	74.3	135.2	126.6	95.7
% children undernourished (weight for age)	53.4	47.0	42.5	56.8	55.9	54.5
% of children with full immunization	35.4	42.0	43.5	24.8	26.4	31.3
% women with anaemia	-	51.8	55.3	-	64.9	68.5
% of deliveries by skilled provider	34.2	42.3	46.6	17.5	23.0	25.4
% of women who have heard of HIV/AIDS	-	40.3	60.9	-	17.2	38.6

Data source: National Family and Health Survey (NFHS-1), 1992-1993; NFHS-2, 1998-1999; NFHS-3, 2005-2006.

Population health interventions include policies or programs - either within or outside of the health sector - that address the underlying causes of ill health embedded in behaviours, contexts and systems of social stratification that increase risk and vulnerability. Population health intervention research encompasses "attempts to capture the value and differential effect of these interventions, the processes by which they bring about change and the contexts within which they work best."[[5], p.I-8] We systematically searched and assessed peer-reviewed literature of population health interventions research involving STs in order to inform policy and to identify important research gaps. The aim of this review was to identify the public health interventions or components of these interventions that are effective in improving population health outcomes (reducing morbidity or mortality rates and reducing risks of ill health) among ST populations in India.

### Scheduled Tribes across India

**Figure 1:**
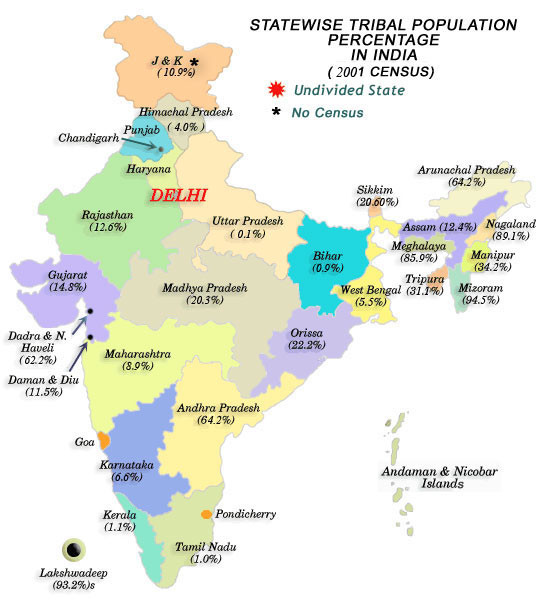
**Map of State wise Tribal Population percentage in India, 2001**. Source: Government of India, 2001.

According to India's most recent census in 2001, there are 84.3 million STs, which is 8.2% of the population [6]. The distributions of ST populations varies widely across India's states and territories. (Figure 1) In Mizoram and Lakshadweep, STs represent close to 95% of the population whereas in Kerala and Tamil Nadu STs represent only 1% of the population. Among the total ST population in India, the highest proportions are found in Madhya Pradesh (14.5%), Maharashtra (10.2%), and Orissa (9.7%). There are around 700 different tribes living across India, predominantly in remote areas: forests, hills, and rough terrain in plateau areas [3]. There is great heterogeneity across different tribal groups, including a sub-category of particularly vulnerable STs known as "primitive" tribes (due to the derogatory nature of this term, these groups are being renamed by the Government of India as 'particularly vulnerable tribal groups') [3].

### The health of Scheduled Tribes

ST populations continue to carry high burdens of 'diseases of the poor', namely undernutrition and infectious diseases. High levels of chronic undernutrition have been observed among child and adult populations [7]. Micronutrient malnutrition is also a major problem among STs, including anaemia and iodine deficiency disorders [8-10]. Malaria persists, particularly among tribal populations living in forested areas and the prevalence has been found to be rising in some areas [11,12]. Prevalence of tuberculosis varies across tribal populations. A number of studies have found that the prevalence and patterns of TB does not differ significantly from non ST communities, but that TB control programs for STs require special attention due to difficult terrain and limited drug supplies in many tribal areas [13-16]. Geographical isolation and limited interactions with other communities has limited exposure of HIV/AIDS among ST communities and among some tribal groups prevalence rates remain low;[17] however in some areas, STs are emerging as a high-risk group for HIV/AIDS as they migrate driven by displacement or for employment opportunities [18].

STs face a number of risks of ill-health including high rates of poverty, illiteracy, harsh living environments, high rates of smoking and alcohol use, and poor access to health care [2,3,19-23]. ST communities have also faced high levels of discrimination, displacement and alienation from their land and livelihoods [3]. In the case of previously enslaved tribal groups, there is evidence suggesting high levels of resignation among STs and limited capacity to take advantage of benefits available to them, reducing their opportunities for good health [24,25]. While historically gender relations among STs were more egalitarian compared to other social groups, anti-female patterns of discrimination have been increasing among some tribal communities as their lives become integrated in mainstream culture and social practices, generally through the conversion to Hinduism [26,27].

### Research and Scheduled Tribes

In other contexts, indigenous populations have been harmed through inappropriate research methods and practices [28-30]. ST communities are also vulnerable to this, due to their high levels of impoverishment, exposure to discrimination, diverging worldviews from other social groups, and lack of voice to express their own priorities and views. What matters is not only to pursue research to improve the health of indigenous populations, but how this knowledge is gathered. Research should be undertaken in a manner that is culturally sensitive and considers the needs, priorities, and 'ways of knowing' of ST populations. To integrate these aspects, there is a need for some level of participation of the ST community, which may include: developing the research question, input in the study design and interpretation of study findings, and developing recommendations for policy. In this paper, we pay particular attention to the considerations that researchers have taken with respect to the nature and level of the participation of the ST community in the intervention and research process as well as the ethical components that have been adopted to ensure the protection of the participants.

## Methods

The search was restricted to peer-reviewed articles published in English and French languages during the period of 1990 to 2009. The search was undertaken by the first author during the month of October 2009. Since the health of STs are both a public health issue and a development issue, we searched databases in both the health and social science: PubMed, EconLit, and Social Sciences Index and Abstract. The grey literature was not included for several reasons. First, given India's size and diversity (including languages), it would be extremely difficult to systematically identify and review the literature in a timely manner. Second, as the focus of the review is on intervention research, which requires rigorous methods, the quality of much of the available grey literature would likely be inadequate. Third, in our experience, local nongovernmental organizations have among the greatest expertise in working with ST populations, but they tend not to systematically document their knowledge. Tapping into this evidence base will take innovative approaches (e.g. collaboration of NGOs, ST communities, and academics in writing projects, which was done as part of *Paniya Sadas*, a forum held on marginalised STs in South India) that were beyond the scope of this review.

Four inclusion criteria were to be met for articles to be included in our review. These were: (a) evaluations or intervention studies of a population health intervention, including surveillance systems, health promotion or prevention programs, and treatment programs, (b) undertaken with an ST population or in a tribal area (where the majority of the population have ST affiliations) in India, (c) with a population health outcome(s), which included morbidity or mortality, and risks of ill health and (d) involving primary data collection. Studies that addressed genetic disorders or evaluated a drug or plant for therapeutic properties were excluded. The review of articles proceeded in four steps. First, we conducted our searches using the following abstract key words: (a) Trib*, (b), India (c) health, HIV, tuberculosis, malaria, nutrition, alcohol, tobacco, smok*. Since population health interventions can be defined using a variety of terms, we did not include a term for intervention. We searched the literature for articles that included Trib* (population) and India (setting) and one health outcome, leaving the search for intervention studies in the following step. Second, titles and abstract were screened to meet inclusion criteria, including whether the study assessed a public health intervention. Third, guided by Critical Appraisal Skills Programme (CASP), potential articles were further scrutinised for quality. Any study with a major flaw in design or methodology (the intervention failed to meet either the CASP screening questions or 2 or more of the detailed questions) was excluded. Fourth, the references of the selected articles were reviewed to identify other articles not found in our search. The first author reviewed and prepared critical appraisal summaries for the papers, then, the second author reviewed these summaries, followed by a discussion and synthesis of the findings by both authors.

Information was collected on the description of the study, including the intervention, objectives of the study, region of study, study design, methods and study population (including the specific tribal groups). We further abstracted information on ethical considerations of the study and the nature of ST participation in the research process. Finally, information on the main findings and conclusions were retained.

## Results

**Figure 2:**
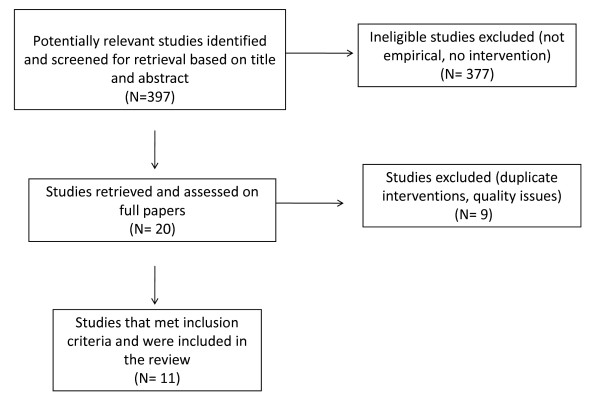
**Search strategy diagram**.

The initial search retrieved 397 potential articles (Figure 2). Following the screening of titles and abstracts, 377 studies were deemed ineligible as they were either not empirical studies that collected primary data or did not assess a public health intervention. This led to 20 potential studies of which the full papers were obtained and reviewed. Nine papers were further excluded due to either poor quality of studies or because there was duplication of interventions by the same authors (we retained the paper which demonstrated the strongest implications for how to build effective public health interventions for ST populations). Our additional search of references from papers retained for our review did not identify any further articles. Therefore, we had a total of 11 papers eligible for the review. Before discussing the findings, we first provide a description of the studies and the extent that ethical considerations and ST participation were integrated in the studies.

### Description of studies

Table 2 presents a description of the studies, which have been published between 1995 (no publications were identified between 1990 and 1994) and 2009. Nine interventions addressed two diseases: the surveillance, prevention, and control of malaria,[31-36] and the control and treatment of TB [37-39]. One study examined an anaemia prevention program [40] and another presented a maternal mortality surveillance system [41]. The studies were conducted predominantly in either tribal states or states with significant proportions of tribes; only 1 study was conducted in a Southern state (Tamil Nadu), where the proportion of tribes is lower. Four studies worked with ST populations exclusively. Studies were undertaken with the Gond tribe, the Savara tribe, the Nicolese, and two studies included multiple tribal groups. The majority of studies, however, did not specify the tribal groups and only one study noted the inclusion of a minority of non-STs within their population sample.

**Table 2:** Description of studies included in the review

First author and year published	Intervention	Region	Tribal areas or populations covered (tribal groups)	Methods	Outcomes
Balasubramanian 1995	Involving ST youth in TB case detection	Tamil Nadu	Tribal hamlets (groups not specified)	10% of general population were randomly sampled and interviewed by medical professionals and performance assessments were conducted by a local team.	No. of TB symptomatic individuals & sputum-positive cases identified.
Singh 2001	Rapid immune-chromatographic test	Madhya Pradesh	Tribal villages (Gond tribe)	Blinded comparison of malaria diagnoses using rapid tests and standard procedures.	Sensitivity, specificity & predictive values of test in detecting malaria.
Murhekar 2004	National Tuberculosis Programme	Car Nicobar	Population (Nicobarese tribe)	Household census. Sputum samples and questionnaire of symptoms. Data compared with survey conducted 15 years ago.	Prevalence of TB infection & smear-positive cases.
Tungdim 2008	Pulmonary TB treatment program	Manipur	Population (Unspecified groups belonging to Mongoloid group)	Cross-sectional study among adult pulmonary TB patients, stratified into: (1) before starting treatment, 2) 2 months of treatment, & 3) completion of treatment. Healthy individuals with no history of TB were matched among non-family members of patients.	Anthropometric measurements (BMI & MUAC) of TB patients.
Barnett 2008	Maternal mortality key informant surveillance system	Jharkhand, Orissa	Clusters with 73% STs (unspecified)	Descriptive study based on existing project data.	No. of births, crude death rates, maternal mortality ratio, causes of maternal death, cost of operating system.
Deshmukh 2008	Adolescent Nutritional Anaemia Project	Maharashtra	Population (unspecified)	Pretest-posttest, comparing haemoglobin estimations, for 3 groups of girls (14-18 years): tribal, rural, urban slums.	Prevalence of anaemia.
Jambulingam 2008	Insecticide treated mosquito nets	Orissa	Tribal villages (Predominantly Kandho, Poroja, Dora, Gadaba, Rana)	PHC records and a series of 5 cross-sectional household surveys (a total of 3,206 households surveyed). Plus bioassays.	Net distribution Acceptance & use of net Retreatment coverage Insecticide persistence
Das 2008	Community-based chloroquine treatment	Orissa	Tribal villages (unspecified)	Compared control and program villages in 1^st^, 2^nd^, 3^rd^, year of operation.	Fever incidence Malaria parasite incidence Parasite prevalence
Prakash 2008	Insecticide treated mosquito nets	Nagaland, Mizoram, Assam	Tribal villages (Zeilian, Ao, Mizo, Kachari-Ahom)	Cross-sectional household surveys in ITN (N = 899) and non-ITN sites (N = 448)	Net use, beliefs & practices
Srivastava 2009	GIS based approach to surveillance of malaria hotspots	Madhya Pradesh	Tribal districts & blocks (unspecified)	Descriptive study based on existing project data.	Annual parasite incidence
Gunasekaran 2009	Long lasting insecticide treated mosquito nets	Orissa	Tribal districts	Compared villages with and without ITN, stratified by high/low endemicity. Survey (908 households) and qualitative methods (Key informant interviews, focus groups).	Net use & beliefs Willingness to pay for nets

No experimental designs were identified; the majority of studies were quasi-experimental, one of the quasi-experimental studies include a qualitative component [35]. Participatory research approaches were not used and gender analysis was not incorporated into any of the studies. No study included a theoretical input to guide their study. Outcomes assessed by the studies included measures related to the uptake of the intervention (e.g. use of bed nets) and health outcomes (e.g. prevalence of malaria).

### Ethics and ST participation in studies

Eight studies did not make any statement regarding ethics. One study cited that they had obtained ethical approval from a scientific research centre [31]. Informed consent was collected in three studies, [31,35,38] although only one study provided details on how informed consent was collected [35]. We identified no study that had specific statements regarding the participation of ST populations in the research process. In two studies, the authors described community meetings that were held prior to the implementation of the intervention to explain the purpose of the ITN program and how to use the ITNs [33,35] In another study community meetings were held to explain the program and to request cooperation from the population [38]. Two studies integrated ST participation *as part of the intervention *(described below), but none of the studies stated any involvement of STs in developing the research question, providing input on the study design, assisting in interpreting the findings or developing policy recommendations.

### Findings

#### Low-cost, rapid results, and easily administered

Several authors stressed the need to provide interventions that are relatively low-cost, demonstrate rapid results, and easily administered. In prevention efforts, three studies examining insecticide-treated nets (ITNs) found that ITNs could be easily distributed, were widely accepted and used by the community, and provided a mass protective effect against malaria [32,33,35] However, challenges arose when participants were required to retreat the nets at their own costs.

Regarding diagnostics, one study proposed that the rapid immune-chromatic test, which was demonstrated to be sensitive (91%) and specific (80%), was particularly effective for ST communities living in forested areas because this test is easier and faster to perform than microscopy [31]. The test does not require laboratory or technical equipment, a simple diagnostic facility can be set up and can be performed by unskilled personnel. Moreover, the test provides immediate results (as opposed to delays generally associated with medical laboratories), therefore, a physician can quickly communicate to their patients the results and begin appropriate treatment.

Of the two surveillance studies, one examined a prospective maternal mortality surveillance system that used key informants, in contrast to retrospective systems used in most low-income settings and relying upon secondary data sources. The surveillance system, which covers three districts and a total population of 228, 186, was found to produce reliable data at a relatively low cost (386 USD per month) [41]. A second surveillance system used a Geographic Information System (GIS) to generate dynamic maps of hot spots for malaria in tribal areas at the district and village levels, with the purpose of developing rapid response for malaria control [36]. Using the GIS approach, map-generated figures can be easily and quickly updated and information can be done through electronic formats - computer facilities are located at the district level. Furthermore, once it is set up, the system can be easily converted to monitor other diseases, such as dengue. No cost estimates of this intervention were provided.

#### Multi-pronged approaches

In order to increase the effectiveness of interventions suggestions were made for multi-pronged approaches. Two studies found that the widespread acceptance of long lasting insecticide treated mosquito nets (LLINs) was often not due to an understanding of the link between their use and malaria prevention but for other reasons, such as limiting the nuisance of mosquito bites [33,35] The authors suggest that the distribution of LLINs is insufficient without adequate transfer of knowledge of malaria and how it is spread, and recommend an educational component to coincide with the introduction of LLINs programs. Another study found that girls aged 14 to 18 years who participated in a program that combined iron-supplementation with a life-skills training program (that included the importance of consuming iron for their health) found a significant improvement in their haemoglobin levels [40]. This study, however, did not separately examine the effects of the iron-supplementation and the training program. Therefore, it cannot be determined the effect, if any, of the training program on reducing haemoglobin levels of participants. One study demonstrated the improvement in nutritional status of STs with TB following the beginning of their treatment [39]. Given that malnutrition predisposes individuals to TB, addressing both malnutrition and TB will be more effective that addressing either factor in isolation.

#### ST participation

Integrating ST participation into public health interventions was proposed in several studies. One study trained literate ST youth volunteers in detecting cases of pulmonary TB in their communities [37]. The training occurred over a relatively short time and engaged 61 male unemployed ST youths who diagnosed TB patients as effectively as skilled para-medical workers from the same area (misdiagnosis was 2%). Another study trained village volunteers in a tribal area (although we could not determine if these volunteers had ST affiliations) to distribute chloroquine to malaria patients and to fill out a 'fever treatment sheet' upon each deliver [34]. Volunteers were selected by the villages (or heads of villages) and were generally male and either small farmers or agricultural labourers. About 10% of the volunteers were replaced due to loss of interest or poor performance, but the majority continued, enjoying the 'social recognition' of delivering the treatments. Over a period of 3 years, the volunteers treated 88,575 fever cases, and malaria mortality and morbidity was reduced significantly more in villages with the program compared to control villages. These results need to be interpreted with caution, however due to potential selection bias; the authors controlled for prevalence of malaria in the villages, but other factors that may impact outcomes of their intervention (e.g. poverty levels, literacy rates, use of bednets) were not addressed. Community-involvement in an ITN program was also found to increase the likelihood of the retreatment of the nets therefore preserving their protective effect from malaria [32].

## Discussion

Despite the large disparities in health between ST and non ST populations in India, we identified only a small number of articles that examined interventions to improve the health of STs. Furthermore, the studies in this review included only disease-specific interventions; we did not identify any article that assessed a comprehensive health intervention for STs. Finally, assessing public health interventions requires rigorous methods and the majority of studies were either descriptive or had a weak study design, even after rejecting those that were more seriously limited. Implementing stronger quasi-experimental designs that address major biases, such as selection bias, would have provided stronger evidence [42]. However, experimental and quasi-experimental designs may not be feasible or appropriate to assess some interventions, notably complex interventions with multiple interacting components [43]. Researchers need to consider various 'trade-offs' that may arise in the pursuit of the best choice of design, given the type of intervention, the populations, the context, and the availability of resources [43,44]. But regardless of study design, there are steps researchers can take to ensure their approach is rigorous and the study is of good quality [45]. In our review, we found the evidence collected to be limited in its quantity, scope, and methodological rigour, and thus should be interpreted with caution. Reviews of the effectiveness of public health interventions in other contexts with indigenous populations have similarly found an 'underdeveloped' evidence base in terms of both the number of available studies and the rigour of the studies [46-48].

The evidence compiled in this review revealed three issues that promote effective public health interventions with STs. First, there is a need to develop and implement interventions that are low-cost, give rapid results and can be easily administered. This addresses the challenge of delivering effective interventions to ST communities who predominantly live in remote areas, where there is limited access to health care, few laboratory facilities, inadequate surveillance, poor vital registrations, and few skilled workers to implement highly technical tasks. Innovative technologies, such as rapid diagnosis tests that do not require laboratories and GIS, offer new opportunities. But by moving one step further and connecting innovative technologies with ST culture and harnessing STs' capacity to use these technologies, could increase opportunities for STs to develop new skills and increase their control over interventions.

Second, even though the study included interventions that addressed a specific disease or a single health need, a multi-pronged approach was advanced by several authors based largely on limitations to the intervention design identified by their studies. ST populations face multiple risks of ill health and are exposed to a number of diseases. Combining two or more activities in a single intervention will likely improve health outcomes. This has been demonstrated among other indigenous groups and vulnerable populations in low-income countries [49]. Given the high levels of health need there is a need for a public health (as opposed to a medical or disease approach) to improve the health of STs and reduce the disparities in health that exist between STs and other social groups. For policy-makers considering new intervention designs, the challenge is to retain the 'quick-wins' of low-cost and easy administration, but always with an eye to adding more elements to a program to make it more comprehensive and capable of responding to health determinants residing in the contexts of STs' cultural, geographic and economic environments.

Third, the involvement of ST populations in the intervention was advocated to help address the isolation of many ST communities, the cultural specificities of STs, the limited resources available and to promote community control. Involving ST populations may improve current programs that have demonstrated poor performance. For example, an increase in the prevalence of TB between 1986 and 2002 was observed in Car Nicobar (the administrative headquarters for the Nicobar district in the Andaman and Nicobar Islands), despite the implementation of a national TB program [38]. The authors argue that this increase may be attributed to the lack of a district level TB control program which contributed to insufficient TB control in the area. A district level program as part of the national one could allow for ST participation in its design, improving its community acceptance by, and cultural appropriateness for, the local population. The participation of indigenous populations in public health interventions has been found to be an effective strategy in other contexts [47].

### Future Research

The paucity of good quality evidence on population health interventions for ST populations suggests ways in which the present level of knowledge can be improved. Given the marginalisation and cultural oppression of many ST populations, however, there is also a need to undertake research that is culturally and ethically appropriate. We propose two avenues to pursue this goal. First, the highest ethical standards should be followed. This includes ensuring that individuals and communities are truly informed and consent to the research being undertaken. One approach that has been used with ST populations is the development of an ethical code of research conduct, and collecting community consent prior to individual consent [50]. The development of ethical guidelines undertaking research with ST populations could help to promote national standards. The development of ethical guidelines and tools at the local level could further help to address the needs of specific tribal communities and can be developed in partnership with these communities. Second, indigenous researchers have advocated for a need for new approaches to research that integrate indigenous views and perspectives and promote self-determination, mobilisation and transformation of these communities [28]. One way to pursue these goals is through participatory research, which is rooted in the philosophy that those who are most affected by health and development issues should be active participants in the research process and subsequent policy action [50-53]. Participatory research combines local knowledge, expertise and experiences with scientific methods and theories, and can be used with a range of study designs and methods including randomized controlled trials [52]. The benefits of participatory approaches for intervention research can extend beyond the research project by increasing the capacity of communities to address their own health needs and redress imbalances in power within the community, which may ultimately contribute to reducing social disparities in health [53]. Researchers can also pursue innovative approaches that include developing partnerships with NGOs who have established programs with ST populations in order to document their existent (but largely undocumented) knowledge and other collaborations (e.g. evaluating ST programs implemented by NGOs). Another approach is to develop solid theoretical foundations for public health interventions and health outcomes for STs, which will help to refine hypothesis generating and improve the accuracy of the interpretation of findings [54,55]. These theoretical frameworks can be developed specifically for ST populations, integrating indigenous 'ways of knowing'. Finally, given the need for the implementation of 'low cost interventions', cost effectiveness studies can provide valuable information for decision-making in selecting the most appropriate intervention strategies.

While there is a growing body of knowledge on the health needs of STs, there is a paucity of data on how we can address these needs. This suggests that future research priority be given to quality public health intervention studies that assess a broad range of interventions and outcomes. Key programs to evaluate include: (1) interventions specific for ST populations, both disease-specific interventions and comprehensive tribal health programs, such as the Tribal Health Initiative in South India,[56] (2) tribal development programs that fall outside of the health sector, but address key determinants of health,[57] and (3) population level interventions to identify how these interventions may better address the needs of STs. In the latter case, these interventions should include not only local or national level programs, but also those that address global factors that may impact ST communities, such as climate change, financialization of economic markets and global trade [58,59]. Research should account for heterogeneity that exists across tribal groups (e.g. comparative analyses) and within tribal groups (e.g. gender analysis). Finally, due to the great diversity across India, studies should be undertaken in different contexts (e.g. urban, rural, forested land, etc) across states and territories.

### Policy implications

As India's economy continues to grow and the health of the population improves, there is a need for greater attention and resources to be allotted to those populations who have not benefitted from the country's economic growth and who continue to face high levels of health needs. Despite pursuing affirmative action for more than fifty years, there are persistent gaps in health and well-being between STs and non STs. This suggests the need to devise and implement new policies. Given the large gaps in knowledge on how to improve the health of STs, resources should be targeted to developing a critical mass of researchers in this domain, including training of researchers with ST affiliations.

## Conclusion

The findings of this review identified three effective strategies for improving health outcomes among ST populations in India: low-cost, rapid results, and easily administered programs, multi-pronged approaches, and including ST participation in the intervention. The evidence base, however, is insufficient. There is a need for a better understanding of how to improve their health by pursuing public health intervention research appropriate for ST populations.

## Competing interests

The authors declare that they have no competing interests.

## Authors' contributions

KM conceived the study, undertook the review, and wrote the manuscript. RL guided the study and contributed to the interpretation of the findings and drafting of the manuscript. Both authors reviewed and approved the manuscript.

## Pre-publication history

The pre-publication history for this paper can be accessed here:

http://www.biomedcentral.com/1471-2458/10/438/prepub
